# Cross-sectional study on anxiety in confinement due to covid-19 in a sub-acute and long-stay mental health unit

**DOI:** 10.1192/j.eurpsy.2023.374

**Published:** 2023-07-19

**Authors:** A. Llimona González, J. Mayans Henares, E. Pechuan Martínez, O. Orejas Pérez, C. Masferrer Herrera, C. Muro Celma, L. Vargas Puertolas, J. Peñalver Aguilar

**Affiliations:** ^1^Psychiatry; ^2^Psychology, Parc de salut mar, Barcelona, Spain

## Abstract

**Introduction:**

Chronic psychiatric patients admitted to subacute and long-stay hospital units are especially vulnerable to the situation of confinement due to the pandemic. Throughout 2020 and 2021 they have suffered the consequences of multiple strict confinements given the differences in isolation protocols in hospitalized patients compared to the general population. This has repercussions on the increase in anxious symptomatology, which influences a more torpid and prolonged evolution of mental disorders in this subpopulation.

**Objectives:**

The objective of this study is to study the anxiety levels of patients admitted to a sub-acute and long-stay mental health unit in a situation of confinement due to covid-19.

**Methods:**

We have carried out a cross-sectional descriptive observational study in 25 patients admitted to the subacute and long-stay unit of the Barcelona Forum Center between December 8 and 23, 2021 in the context of confinement due to a covid-19 outbreak. Sociodemographic and clinical variables are collected. We have used the self-administered STAI scale to assess clinical anxiety.

**Results:**

The mean age is 47.7 years; women 60%. 80% with single marital status. 90% of the patients presented active tobacco consumption, with an average of 21.2 cigarettes/day. The mean score on the STAI scale was 58.8 for state anxiety and 46.7 for trait anxiety, both levels above the 75th percentile for adults, both men (state anxiety 28, trait anxiety 25) and women (state anxiety 31, trait anxiety 32).

**Conclusions:**

The state and trait anxiety scores of the STAI scale of hospitalized patients are higher than the average of the general population, which could be due to the situation of confinement due to the covid pandemic.

**Disclosure of Interest:**

None Declared

